# Supplementary material for: Association between plain ropivacaine dose and spinal hypotension for cesarean delivery: a retrospective study
Source: PeerJ. 2024 Oct 23;12:e18398. doi: 10.7717/peerj.18398 (PMC11512552; doi:10.7717/peerj.18398)
Supplement: Supplemental Information 1 [file peerj-12-18398-s001.docx]

**Supplementary Materials**

[File S1: STROBE Statement—Checklist of items that should be included in reports of cohort studies 2](#_Toc167388447)

[Figure S1. Adjusted ORs for incident hypotension 5](#_Toc167388448)

[Figure S2. ROC curve 6](#_Toc167388449)

[Table S1. Association between Plain Ropivacaine Dose and Incident Hypotension (Sensitivity analyses). 7](#_Toc167388450)

# File S1: STROBE Statement—Checklist of items that should be included in reports of *cohort studies*

|  | Item No | Recommendation | Reported on page No |
| --- | --- | --- | --- |
| **Title and abstract** | 1 | (*a*) Indicate the study’s design with a commonly used term in the title or the abstract | Page 2 |
|  |  | (*b*) Provide in the abstract an informative and balanced summary of what was done and what was found | Page 2 |
| Introduction | | |  |
| Background/rationale | 2 | Explain the scientific background and rationale for the investigation being reported | Page 3 |
| Objectives | 3 | State specific objectives, including any prespecified hypotheses | Page 3 |
| Methods | | |  |
| Study design | 4 | Present key elements of study design early in the paper | Page 4-5 |
| Setting | 5 | Describe the setting, locations, and relevant dates, including periods of recruitment, exposure, follow-up, and data collection | Page 4-5 |
| Participants | 6 | (*a*) Give the eligibility criteria, and the sources and methods of selection of participants. Describe methods of follow-up | Page 4-5 |
|  |  | (*b*) For matched studies, give matching criteria and number of exposed and unexposed | n/a |
| Variables | 7 | Clearly define all outcomes, exposures, predictors, potential confounders, and effect modifiers. Give diagnostic criteria, if applicable | Page 4-5 |
| Data sources/ measurement | 8* | For each variable of interest, give sources of data and details of methods of assessment (measurement). Describe comparability of assessment methods if there is more than one group | Page *4-5* |
| Bias | 9 | Describe any efforts to address potential sources of bias | n/a |
| Study size | 10 | Explain how the study size was arrived at | Page 5 |
| Quantitative variables | 11 | Explain how quantitative variables were handled in the analyses. If applicable, describe which groupings were chosen and why | Page 5 |
| Statistical methods | 12 | (*a*) Describe all statistical methods, including those used to control for confounding | Page 5 |
|  |  | (*b*) Describe any methods used to examine subgroups and interactions | Page 5 |
|  |  | (*c*) Explain how missing data were addressed | Page 5 |
|  |  | (*d*) If applicable, explain how loss to follow-up was addressed | Page 5 |
|  |  | (*e*) Describe any sensitivity analyses | Page 5 |
| Results | | |  |
| Participants | 13* | (a) Report numbers of individuals at each stage of study—eg numbers potentially eligible, examined for eligibility, confirmed eligible, included in the study, completing follow-up, and analyzed | Figure 1 |
|  |  | (b) Give reasons for non-participation at each stage |  |
|  |  | (c) Consider use of a flow diagram |  |
| Descriptive data | 14* | (a) Give characteristics of study participants (eg demographic, clinical, social) and information on exposures and potential confounders | Table 1and 2 |
|  |  | (b) Indicate number of participants with missing data for each variable of interest | Figure 1 |
|  |  | (c) Summarise follow-up time (eg, average and total amount) | Page 4 |
| Outcome data | 15* | Report numbers of outcome events or summary measures over time | Page 6 |
| Main results | 16 | (*a*) Give unadjusted estimates and, if applicable, confounder-adjusted estimates and their precision (eg, 95% confidence interval). Make clear which confounders were adjusted for and why they were included | Page 6 |
|  |  | (*b*) Report category boundaries when continuous variables were categorized | Page 5-6 |
|  |  | (*c*) If relevant, consider translating estimates of relative risk into absolute risk for a meaningful time period | n/a |
| Other analyses | 17 | Report other analyses done—eg analyses of subgroups and interactions, and sensitivity analyses | Page 6 |
| Discussion | | |  |
| Key results | 18 | Summarise key results with reference to study objectives | Page 7 |
| Limitations | 19 | Discuss limitations of the study, taking into account sources of potential bias or imprecision. Discuss both direction and magnitude of any potential bias | Page 8 |
| Interpretation | 20 | Give a cautious overall interpretation of results considering objectives, limitations, multiplicity of analyses, results from similar studies, and other relevant evidence | Page 7-8 |
| Generalisability | 21 | Discuss the generalisability (external validity) of the study results | Page 8 |
| Other information | | |  |
| Funding | 22 | Give the source of funding and the role of the funders for the present study and, if applicable, for the original study on which the present article is based | Title page |

# Figure S1. Adjusted ORs for incident hypotension


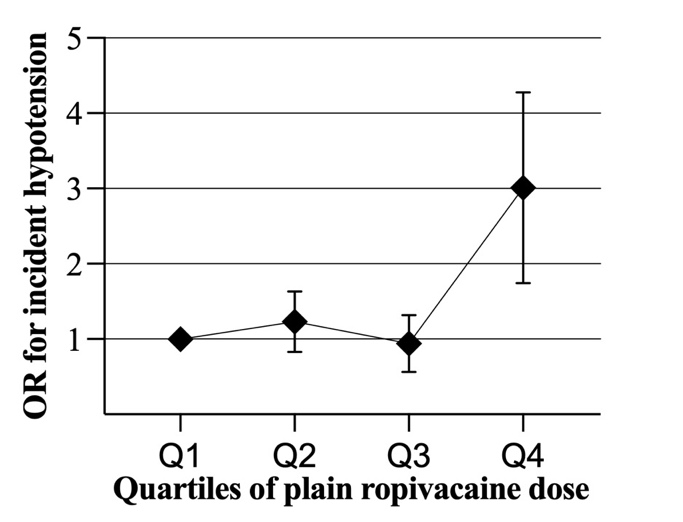


**Supplemental Figure 1** Association between the plain ropivacaine dose and incident hypotension. ORs were adjusted for maternal age, maternal weight, gestational age, hypertension, singleton or multiple pregnancies, planned or emergency surgery, puncture location (L2/3 or L3/4), anesthesiologist seniority, anesthesia to incision time, and anesthesia to delivery time. Q1 to Q4 represent the quartiles of the plain ropivacaine dose. OR: Odds ratio.

# Figure S2. ROC curve


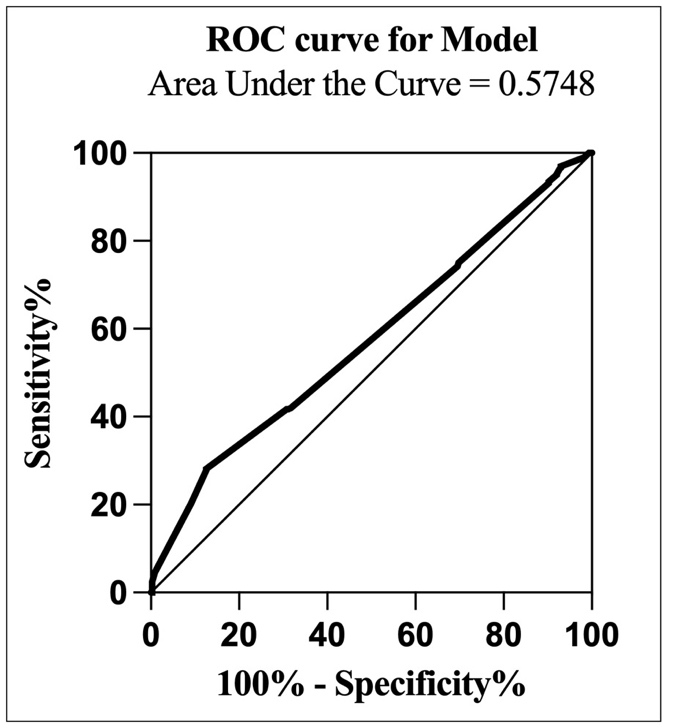


ROC curve for the association between the plain ropivacaine dose and incident hypotension. ROC: Receiver operating characteristic.

# Table S1. Association between Plain Ropivacaine Dose and Incident Hypotension (Sensitivity analyses).

| **Supplemental Table 1** Association between Plain Ropivacaine Dose and Incident Hypotension (Sensitivity analyses). | | | | | | | | | |
| --- | --- | --- | --- | --- | --- | --- | --- | --- | --- |
|  | OR (95CI) | | | | | *P* Value for Trend |  |  |  |
|  | Q1^a^  (15.0 [≤15.75]) ^a^ | | Q2 ^a^  (16.5 [15.76–16.5]) ^a^ | Q3 ^a^  (17.25 [16.51–17.25]) ^a^ | Q4 ^a^  (18.75 [≥17.26]) ^a^ |  |  |  |  |
| Model S0, Data included  in the study ^b^ (N=1182) | | | 1.0 | 1.12 (0.85, 1.65) | 0.89 (0.59, 1.34) | 2.83 (1.84, 4.36) | < 0.001 |  |  |
| *P* Values* | | |  | 0.31 | 0.58 | < 0.001 |  |  |  |
| Model S1, Multiple imputation ^c^ (N=1219) | | | 1.0 | 1.19 (0.86,1.65) | 0.86 (0.58,1.28) | 2.74 (1.80, 4.18) | < 0.001 |  |  |
| *P* Values* | | |  | 0.28 | 0.47 | < 0.001 |  |  |  |
| Model S2, Excluding hypertensive patients ^d^ (N=1128) | | | 1.0 | 1.26 (0.85, 1.65) | 0.90 (0.60, 1.37) | 2.92 (1.88, 4.53) | < 0.001 |  |  |
| *P* Values* | | |  | 0.18 | 0.63 | < 0.001 |  |  |  |
| ^a^ Q1, Q2, Q3, Q4 are quartiles of plain ropivacaine dose (mg, median [range]). ^b^ Model S0 data is Model 2 data in Table 3. ^c^ Model S1 was adjusted for the confounding factors of Model 2 after Multiple imputations for missing data. ^d^ Model S2 was also adjusted for the confounding factors of Model 2 after excluding patients with pregnancy-induced hypertension. * P Values in Q2, Q3, and Q4 are all compared to Q1. Abbreviation: OR, Odds Ratio; CI, Confidence Interval. | | | | | | | | | |
